# *Drosophila melanogaster*: A Model Organism to Study Cancer

**DOI:** 10.3389/fgene.2019.00051

**Published:** 2019-03-01

**Authors:** Zhasmine Mirzoyan, Manuela Sollazzo, Mariateresa Allocca, Alice Maria Valenza, Daniela Grifoni, Paola Bellosta

**Affiliations:** ^1^Department of Cellular, Computational and Integrative Biology, University of Trento, Trento, Italy; ^2^Department of Pharmacy and Biotechnology, University of Bologna, Bologna, Italy; ^3^Department of Biosciences, University of Milan, Milan, Italy; ^4^Department of Medicine, NYU Langone Medical Center, New York, NY, United States

**Keywords:** *Drosophila cancer modeling*, cancer biology, oncogene, tumor suppressor, tissue growth, signaling, metabolism, therapeutic approaches

## Abstract

Cancer is a multistep disease driven by the activation of specific oncogenic pathways concomitantly with the loss of function of tumor suppressor genes that act as sentinels to control physiological growth. The conservation of most of these signaling pathways in *Drosophila*, and the ability to easily manipulate them genetically, has made the fruit fly a useful model organism to study cancer biology. In this review we outline the basic mechanisms and signaling pathways conserved between humans and flies responsible of inducing uncontrolled growth and cancer development. Second, we describe classic and novel *Drosophila* models used to study different cancers, with the objective to discuss their strengths and limitations on their use to identify signals driving growth cell autonomously and within organs, drug discovery and for therapeutic approaches.

## Introduction

**Figure 1:**
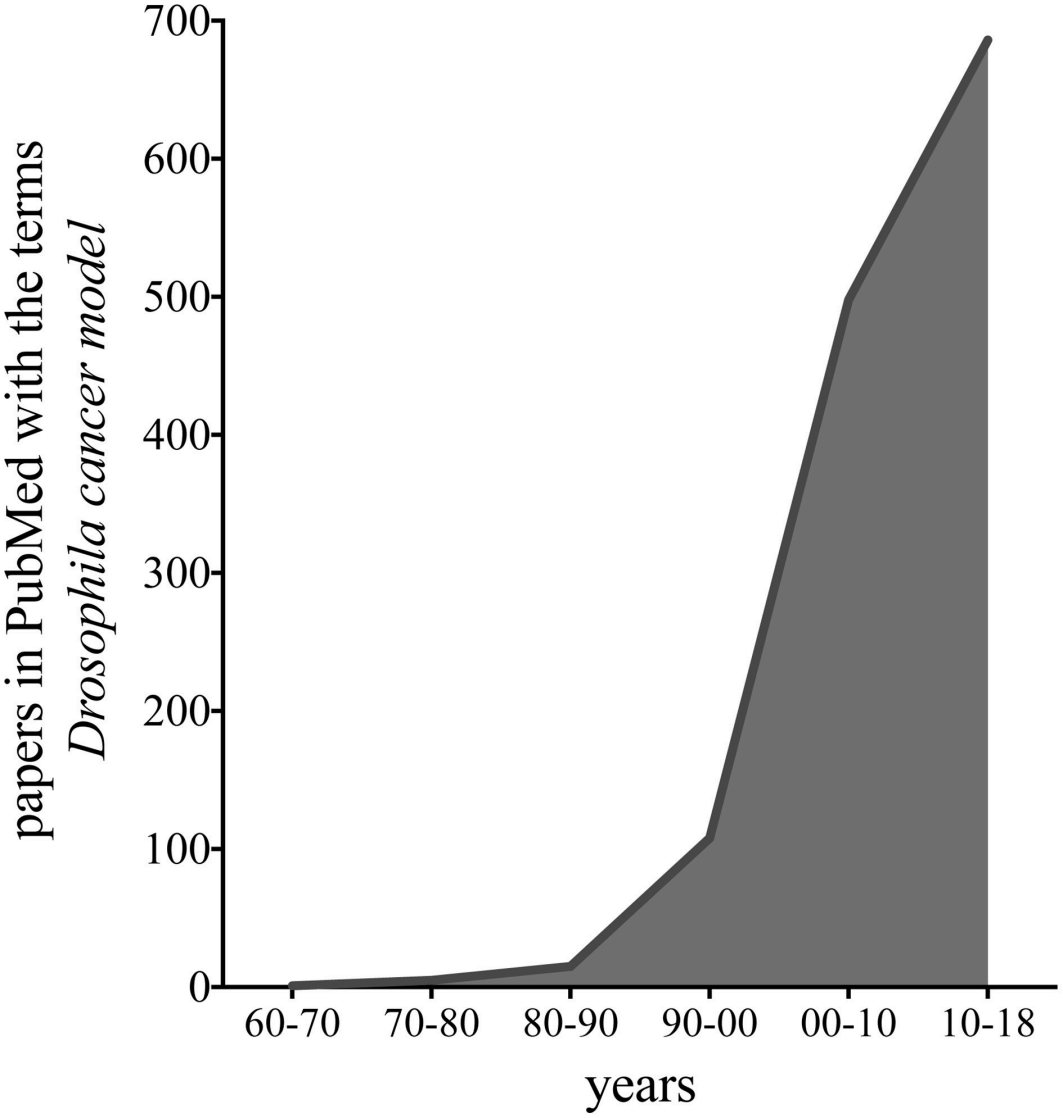
Graph representing the number of publications in PubMed found with the terms “*Drosophila* cancer model,” in the last 48 years.

The fruit fly, *Drosophila melanogaster*, is used as a model organism to study disciplines ranging from fundamental genetics to the development of tissues and organs. *Drosophila* genome is 60% homologous to that of humans, less redundant, and about 75% of the genes responsible for human diseases have homologs in flies (Ugur et al., 2016). These features, together with a brief generation time, low maintenance costs, and the availability of powerful genetic tools, allow the fruit fly to be eligible to study complex pathways relevant in biomedical research, including cancer. Indeed, publications that use flies to model cancer have exponentially increased in the last 10 years, as shown in the graph of Figure 1, suggesting the relevance of this model to cancer research.

In this review we first describe the basic biological mechanisms responsible for uncontrolled growth conserved between humans and flies. We placed a particular emphasis on the characterization of epithelial tumors from most studied models (gut and brain), to novel approaches for studying tumor-induced angiogenesis, prostate, thyroid and lung cancers, with the goal to discuss their strengths and limitations. In the second part, we analyze few physiological mechanisms that uncover potential non-autonomous mechanisms controlling growth, including the relation between the immune cells (macrophages) and the growth of epithelial cells, or the function of lipid metabolism in cancer growth. Finally, we discuss how *Drosophila* models are used to find novel interesting therapeutic approaches.

## Properties of Epithelial Cancer Cells

Cancer cells are characterized by unrestrained proliferation that results from defects in signaling driving cellular growth, apoptosis and changes in metabolic pathways. At cellular level, the hyperproliferative status of cancer cells is mainly due to the activation of growth signals induced by proto-oncogenes (e.g., the RAS/RAF/MAPK axis), which function downstream of receptor signaling cascades, and are deregulated in 25% of human tumors (Samatar and Poulikakos, 2014). Tumor cells escape the anti-proliferative effect of tumor suppressor genes, such as *RB* (retinoblastoma-associated) and *TP53* genes (Duronio and Xiong, 2013), through mutations in these genes, which result in uncontrolled growth (Hanahan and Weinberg, 2000, 2011; Hariharan and Bilder, 2006). Apoptotic cell death represents another physiological mechanism to maintain cellular homeostasis, and cancer cells have developed strategies to evade apoptosis, i.e., by increasing the activity of anti-apoptotic genes (*Bcl-2, Bcl-xL, Bcl-w*) and of pro-survival factors (*Igf-1, Igf-2*) or by downregulating the action of pro-apoptotic genes (*Bax, PUMA, Bin*) (Hanahan and Weinberg, 2011). Another characteristic of cancer cells is the reactivation of telomerase, present in 90% of human cancers, that allows them to replicate unlimitedly (Kumar et al., 2016).

Cancer cells also exhibit alterations in metabolic pathways that contribute to their survival. Rapidly proliferating cells have a high metabolic rate and suffer from low oxygen conditions (hypoxia). In epithelial tumors, this condition triggers the so-called angiogenic “switch” where the quiescent vascular network is induced to proliferate by the secretion of pro-angiogenic factors, such as VEGF (Vascular Endothelial Growth Factor) and FGF (Fibroblast Growth Factor) (Hida et al., 2018), allowing the formation of new vessels that penetrate into the tumor mass to supply oxygen and nutrients (Carmeliet and Jain, 2011). Cancers cells also exhibit a metabolic switch where they reprogram their metabolism to use an alternative and less abundant anabolic pathway to sustain their growth. In particular they switch from oxidative phosphorylation to anaerobic glycolysis, where glucose is used to produce lactate, through a process called the “Warburg effect” (Pavlova and Thompson, 2016; Vander Heiden and DeBerardinis, 2017). This metabolic switch is not yet completely characterized but is supported by the activation of oncogenes, including Myc that also activates glutaminolysis to fuel the TCA cycle with anaplerotic reactions to produce the intermediates necessary for cellular biosynthesis (Hsieh and Dang, 2016).

The last stage of tumorigenesis is represented by the invasive and metastatic capabilities of tumor cells to disrupt the apical-basal cell polarity, a process that is associated with the downregulation of cell-cell contact molecules and the release of metalloproteases (MMP1), lytic enzymes that degrade the extracellular matrix (ECM) allowing tumor cells to escape and colonize an environment that suites them and to acquire new oncogenic properties (Massague and Obenauf, 2016; Lambert et al., 2017). A variety of studies are now focused on how the tumor micro environment (TME), a specific niche composed of fibroblasts, lymphocytes and immune cells, that may shape pre-cancer cells for their progression into cancer cells and it may select the development of metastasis (Massague and Obenauf, 2016). Emergent evidence suggests also a key role for non-autonomous signals released by the cells composing the niche, particularly from cancer-associated fibroblasts (CAFs), that are essential to support the growth of cancer cells in this “new” metabolic environment (Lambert et al., 2017).

## Cancer Modeling in *Drosophila*

Most of the signaling pathways controlling cell growth and invasion in mammals have a conserved function in flies allowing their modulation into models that mimic tumor's biology in a simple model organism like *Drosophila* (Millburn et al., 2016). The combination of genetic screens with the availability of powerful recombination techniques enabled also a rapid characterization of the primary function of conserved oncogenes and of tumor suppressor genes in a whole animal (Sonoshita and Cagan, 2017). In addition, recent studies using *Drosophila* imaginal discs explored the mechanisms that govern growth in epithelial tumors and their interaction with the local TME and stromal cells, including some steps in the recruitment of the immune cells (macrophages) to the tumor mass (Herranz et al., 2016; Muzzopappa et al., 2017).

## Epithelial Tumors in *Drosophila*

**Figure 2:**
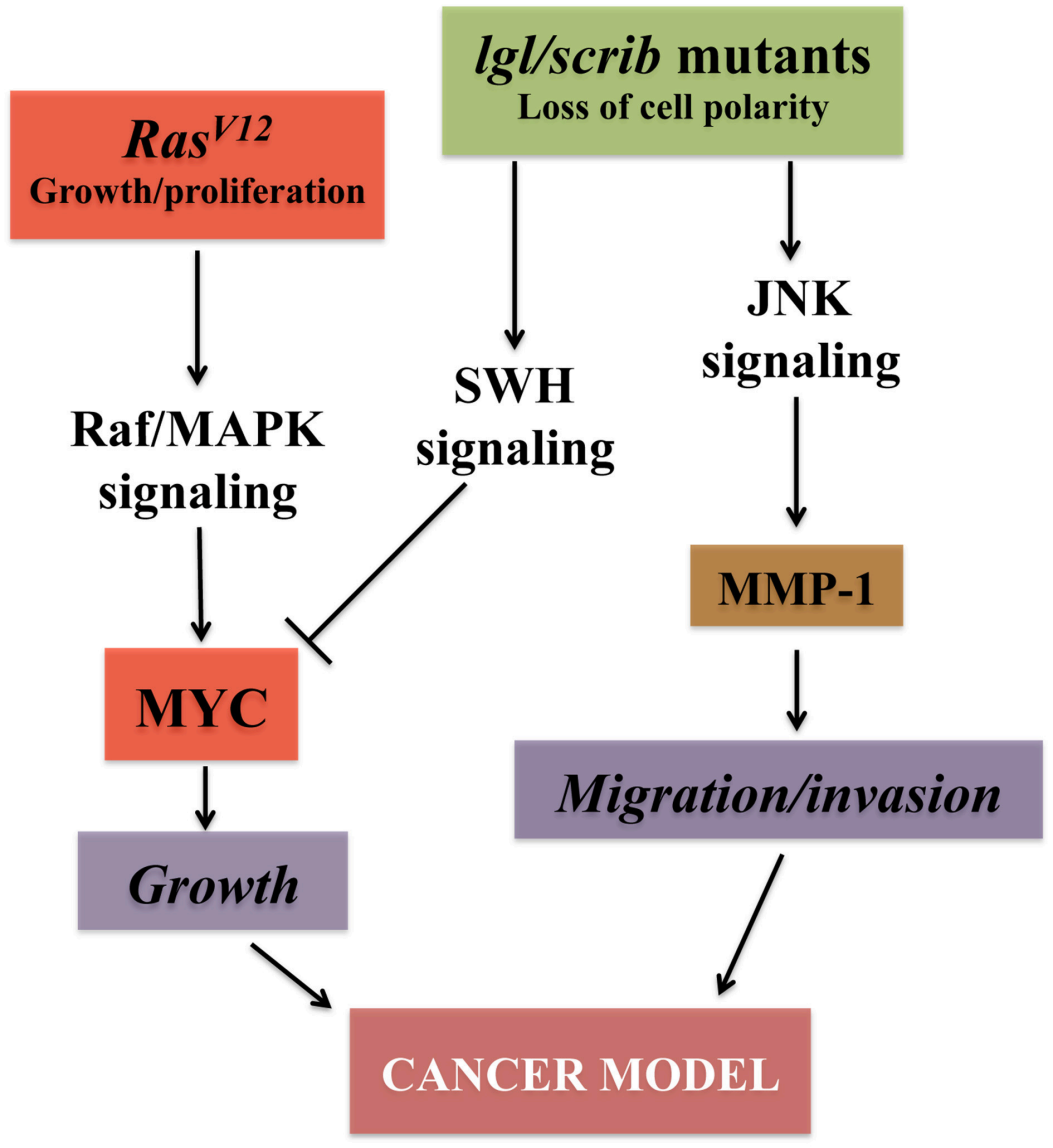
Major pathways converging on uncontrolled growth in *Drosophila* epithelial cells. The signaling pathways outlined confer growth, migration and invasive capabilities to epithelial cells both in vertebrates and flies. Models that mimic the growth of epithelial cancer cells and their ability to undergo metastasis in *Drosophila* have been established by inducing the cooperation between oncogenes (RED) like the active form of Ras (*Ras*^*V*12^) together with the loss of function of cell polarity genes (GREEN) (Brumby and Richardson, 2003; Pagliarini and Xu, 2003). Alteration of cell polarity with the downregulation of the SWH (Salvador-Hippo-Warts) pathway, together with *Ras*^*V*12^, triggers downstream events, including activation of the MAPK signaling that stabilize Myc protein (Galletti et al., 2009) resulting in robust cellular growth. Activation of the JNK signaling, with the concomitant loss of cell polarity, induces metalloproteases (MMP-1) and confers to the epithelial cells the distinct characteristics of migration and invasion, hallmarks of tumor growth (Uhlirova et al., 2005; Igaki et al., 2006; Ma et al., 2017).

About 90% of human cancers are of epithelial origin (Hanahan and Weinberg, 2000). Epithelial tissues are characterized by a specific cell architecture composed of junctions and apical and baso-lateral membrane domains that are crucial for the maintenance of cell-physiological functions. Loss of cell adhesion and cell polarity, with an increase of cell motility, are indeed characteristic early cancer traits. In this context, *Drosophila* larval imaginal discs are a monolayer epithelium that is limited apically by a squamous epithelium (peripodial membrane) and, basally to the notum, by a layer of myoblasts embedded in Extracellular Matrix, and constitute a perfect system in which to model the onset of epithelial cancer progression. These larval organs are indeed morphologically and biochemically comparable to mammalian epithelia (Wodarz and Nathke, 2007). Moreover, the prominent signaling pathways that regulate growth in humans are conserved in the fruit fly (Figure 2), allowing the use of this animal model to examine the hallmarks of cancer (St. Johnston, 2002). During the last few years, the imaginal wing and eye discs have been used successfully to study tumor growth and invasion, to investigate the function of cancer genes, and to perform chemical screenings (Tipping and Perrimon, 2014). The imaginal discs also represent an excellent model to analyze oncogenic cooperation: thanks to the use of the MARCM system (Lee and Luo, 1999), it is feasible to induce simultaneously in single cells mutations in tumor suppressor genes (e.g., mutations in cell polarity genes and Hippo pathway components and interactors) and oncogenic activating mutations, or to overexpress specific genes (e.g., EGFR, Ras, Myc, Yorki), resulting in tissue overgrowth, alteration of the normal tissue architecture, disruption of the basement membrane, and invasive/metastatic behavior (Brumby and Richardson, 2003; Pagliarini and Xu, 2003; Wu et al., 2010).

### Marks of Alteration in Epithelial Cells

#### Loss of Cell Polarity

Cellular junctions and a proper apical-basal cell polarity are fundamental for the maintenance of epithelial tissue architecture and function. During early cancer stages, tissues lose these properties and cells subvert their normal growth rate and acquire invasive and migratory behaviors (Wodarz and Nathke, 2007; Bryant and Mostov, 2008). In *Drosophila*, three complexes establish and maintain epithelial polarity: the Crumbs/Stardust/PATJ/Bazooka, the Par6/aPKC (atypical protein kinase-C) and the Scrib/Dlg/Lgl (Scribble/Discs large/Lethal giant larvae) complexes, which are respectively placed at the apical, subapical and baso-lateral membrane domains. Alterations in these proteins provoke continued cell proliferation, loss of differentiation and complete loss of tissue architecture, resulting in neoplastic overgrowth (Bilder, 2004; Grzeschik et al., 2010; Johnson and Halder, 2014). *lgl* was the first neoplastic tumor suppressor gene discovered in *Drosophila* and its loss leads to an abnormal growth of the imaginal structures and the larval brain. In addition, *lgl* mutant tissues, and tissues bearing *dlg* or *scrib* mutation, have the ability to form secondary tumors in the thorax, brain, wings, muscles, intestine and ovaries (Woodhouse et al., 1998). The loss of cell polarity impacts cell proliferation through the deregulation of the Hippo (Hpo) pathway, a signaling cascade involved in organ size maintenance (Lu et al., 2010). It is not yet fully known how *lgl* activity interacts with the Hpo cascade, but it was observed that its downregulation up-regulates cell cycle genes (such as Cyclin E and E2F1) (Grzeschik et al., 2007) and permits the nuclear translocation of Yorkie (Yki), the downstream effector of the Hippo pathway, causing the activation of its target genes, including MYC, that was found to be important for the growth of *lgl* mutant clones in a competitive environment (Froldi et al., 2010). In humans, two *lgl* homologs have been discovered, *HUGL-1* and *HUGL-2*, with *HUGL-1* rescuing all the defects of the fly *lgl* mutant (Grifoni et al., 2004). *HUGL-1* loss of function has been associated with a series of human malignancies (Schimanski et al., 2005; Grifoni et al., 2007; Lu et al., 2009). Finally, while the human genome encodes for only one homolog of the tumor suppressor *scrib*, a number of homologs are known for *dlg* which have been implicated in different types of cancer (Halaoui and McCaffrey, 2015).

#### Growth Signaling

The **Salvador-Warts-Hippo (SWH)** tumor suppressor pathway was discovered first in *Drosophila* as a regulator of organ size (Pan, 2010; Yu et al., 2015) and later in humans, where it was found to be fundamental in the regulation of cancer growth (Harvey et al., 2013). The physiological activation of the Hippo (HPO) kinase, (MST1/2 in human) (Harvey et al., 2003) consists in the phosphorylation of Warts (WTS), (LATS1/2 in human) (Genevet et al., 2010; Yu et al., 2010) and in the activation of the phosphorylated core complex, that includes Salvador (SAV in human) (Tapon et al., 2002) and Mob/MATS, that in turn, phosphorylate Yki (YAP/TAZ in humans) (Oh and Irvine, 2008). Phosphorylated Yki is sequestered and degraded in the cytoplasm, resulting in the inhibition of its nuclear transcriptional activity and oncogenic function (Harvey et al., 2013). Upstream, the Hippo cascade is regulated by components of cell junctions, including cell adhesion molecules such as Merlin, a homolog of the human Neurofibromatosis Type 2 (NF2) (Genevet et al., 2010; Yu et al., 2010), which acts as tumor suppressor; the cadherin Fat in complex with Dachsous; and by cell polarity regulators such as Crumbs (Robinson et al., 2010; Harvey et al., 2013). Alterations in the composition of the core proteins (HPO, WTS, SAV, MATS) of the pathway trigger Yki translocation into the nucleus that binds tissue-specific partners and induces the expression of its target genes, among them: CyclinE, dIAP1 and MYC (Harvey et al., 2003; Pantalacci et al., 2003; Neto-Silva et al., 2010; Ziosi et al., 2010). This articulated system is also tightly regulated by other signaling pathways: for example, in the *Drosophila* imaginal wing disc, Lgl or aPKC deregulation results in JNK activation to promote Yki nuclear translocation via phosphorylation of Ajuba (Jub), an upstream regulator of the cascade that binds to and inhibits Wts kinase activity (Sun and Irvine, 2013). In addition to the regulation of cell-cell interaction signals, components of the Hippo pathway have been found to be sensitive to mechanical stress (Panciera et al., 2017). This mechanotransduction function is critical in the control of physiological pathways, and its deregulation may contribute to the abnormal cell behavior in diseases such as cancer, where the cells in the tumor have to sustain physical forces generated by tissue overgrowth. Interestingly, this last function has shown differences in the behavior of Yki between human and flies: indeed, in *Drosophila* the Yki protein does not respond to integrin stimulation, while in mammals integrin signaling promotes YAP/TAZ activity. One possible explanation for this different behavior may be that the N-terminus of Yki is missing a domain necessary to bind PDZ-containing proteins, which is found in its human counterpart YAP, and is necessary for the activation of the integrin-Src adhesion branch of the pathway (Elbediwy and Thompson, 2018). However, an interesting and potential explanation for this difference comes from a comparative analysis of the Yki protein and the evolution of the different epithelia: this analysis outlines how in *Drosophila* the apical membrane of the columnar epithelium is well differentiated in its function to activate the Hippo pathway, whereas in mammals the multilayer of cells lacks a functional apical domain, and the activation of YAP/TAZ relies on the activation/signal from the integrin adhesion pathways of the stem cells on the basal layer of the epithelium (Elbediwy and Thompson, 2018).

The **RAS/RAF/ERK** signaling cascade is one of the most conserved pathways in all organisms, including *Drosophila*. This pathway is part of the MAP kinase signaling that, in addition to ERK1/2, also includes JNK1/2/3, p38/MAPK, and ERK5, which mainly respond to stress activators (Morrison, 2012). Highly conserved in flies, ERK1/2 are activated by growth factors such as EGF or FGFs. These ligands bind to receptor tyrosine kinases (RTKs) to activate downstream signaling, in particular its core complex, which is represented by the guanidine exchange factor Son of Sevenless (SOS) that, in turn, activates the small G proteins RAS on the cell membrane. This leads to RAF activation and to the formation of the complex with the kinase D-Sor also called MAPKK or MEK that, upon phosphorylation of Rolled, the fly homolog of MAPK or ERK kinases, induces the activation of its final targets (Shilo, 2014). ERK in flies has much fewer targets than those described in vertebrates, the most common being the ETS-domain protein Pointed (Pnt). In particular PntP2, needs to be phosphorylated for its activation and is the principal activator of transcription downstream of many RTKs, and PntP1 is transcriptionally induced by MAPK (Shilo, 2014). A second transcriptional repressor is Capicua (Cic), an HMG box-containing protein highly conserved in vertebrates (Simon-Carrasco et al., 2018). Interestingly, in the last couple of years, this protein was found to possess oncogenic properties and be overexpressed in many tumors (Simon-Carrasco et al., 2018). In addition, Cic activity regulates co-target genes upon Yki activation, placing this protein at the crossroads of RTKs and SWH pathways (Simon-Carrasco et al., 2018).

Even though MAPK targets in *Drosophila* are less abundant than in mammals, its activation and translocation to the nucleus results in a growth phenotype mimicking a few characteristic steps of growth in tumor cells (Brumby et al., 2011). Activation of Ras is considered a cancer distinctive trait both in *Drosophila* and humans, and it represents one of the strategies to model human cancer in flies. In *Drosophila* there are three *Ras* genes but only *Ras1* has functional homology with mammalian *RAS*. In the epithelial cells of the wing imaginal disc, Ras1 activation triggers hyperproliferation but also determines cell fate (Prober and Edgar, 2000). Ras activation is at the crossroads of other growth factor signaling cascades: recently, a link to Hpo function was shown in *Drosophila* epithelial cells, where Ras activation was able to induce the tissues to switch from a pro-differentiative to a pro-growth program by modulating SWH's transcriptional output (Pascual et al., 2017). Ras increases cell proliferation also through the transcriptional regulation of growth factors and their receptors. For example, it helps promote angiogenesis-like mechanisms in tracheal development through secretion of the FGF/EGFR molecules (Petit et al., 2002; Grifoni et al., 2015); its activation stabilizes pro-growth signals including MYC (Prober and Edgar, 2000), and inhibits pro-apoptotic molecules like Hid (Bergmann et al., 1998). Because of all these functional homologies to human RAS, its activation in *Drosophila* is considered a good method to establish models that mimic tumor growth.

The **JNK Signaling Pathway** is activated mainly by oxidative stress, producing reactive oxygen species (ROS), and by Eiger, the *Drosophila* homolog of TNF-α. Its function is variable and depends also on the cellular environment: it can indeed induce cell proliferation and migration, but its major role is to induce apoptosis (Igaki, 2009). The signaling core is characterized by Hemipterus/Hep (JNKK) (Glise et al., 1995), Basket/Bsk (JNK) (Stronach, 2005) and the AP-1 complex, that functions as negative feedback by up-regulating the expression of the Puckered phosphatase (Martin-Blanco et al., 1998). The AP-1 complex is composed of Fra (Fos-Related Antigen) and dJun (*Drosophila* Jun) and is the final effector of the cascade (Kockel et al., 2001). Upstream Hep is phosphorylated by many JNKK kinases (Tak1-12, Mekk1, Ask1, Slpr) and can also be activated by different indirect stimuli (e.g., RAS, JNKKKK/Msn, and Eiger). Cell death is induced by the expression of the pro-apoptotic genes *hid, reaper* and *grim*, whose activity inhibits the pro-survival protein dIAP1 (Weston and Davis, 2007). In *Drosophila* cancer cells, the JNK pathway plays a dual role, by suppressing or promoting growth depending on the context (Brumby and Richardson, 2003; Uhlirova et al., 2005; Cordero et al., 2010). *lgl, scrib*, and *dlg* mutant cells undergo JNK-mediated apoptosis resulting in a mechanism of tumor suppression (Brumby and Richardson, 2003; Uhlirova et al., 2005; Igaki et al., 2006). On the contrary, in tumor cells with active RAS, apoptosis is blocked and JNK signaling acts as a tumor promoter transcribing genes involved in growth and invasion such as MMP1 (Igaki et al., 2006; Uhlirova and Bohmann, 2006). The overexpression of activated RAS together with Hep (*ras*^*v*12^*hep*^*wt*^) gives cells invasive and metastatic abilities, highlighting how these pathways converge to induce transformation in epithelia.

The **PI3K/Target of rapamycin (TOR)** signaling pathway is a highly conserved key regulator of growth. The binding of insulin-like peptides (ILPs) (fly's insulin) to the receptor (InR) results in the phosphorylation of *chico*/IRS1-4, and the production of phosphatidylinositol-3, 4,5-triphosphate (PIP3) by PI3K, a reaction that is counteracted by the lipid phosphatase PTEN (Grewal, 2009). PIP3 recruits several Ser/Thr kinases to the plasma membrane, including Akt/PKB and PDK1 (3′-phosphoinosite-dependent protein kinase-1), while its activation results in the inhibition of Glycogen Synthase Kinase-beta (GSK3-β), a conserved kinase that not only controls energy metabolism by inactivation of Glycogen Synthase, but also regulates Wnt signaling by controlling β-catenin/*armadillo* (Xu et al., 2009) and Myc stability (Bellosta and Gallant, 2010). Activation of Akt also inhibits Tuberous Sclerosis Complex 1 and 2 (TSC1/2), a tumor suppressor binary complex that negatively regulates Rheb, a GTPase upstream of TOR kinase responsible for the activation of TORC1. TOR is found in two complexes: TORC1, which includes Raptor and LST8 adaptor molecules, is sensitive to amino acids and is inhibited by rapamycin; and TORC2, that is composed of LST8 and Rictor adaptor molecules, and does not respond to amino acids or rapamycin (Saxton and Sabatini, 2017). Activation of TORC1 results in phosphorylation of ribosomal protein kinase p-70-S6 (S6K) and of eukaryotic translation initiation factor 4E-binding protein-1(4E-BP1), thereby triggering protein synthesis and initiation of translation. Insulin and TOR activities are also balanced by a negative feedback mechanism that is activated when S6K is hyper-activated to counteract insulin activity. Under this condition, S6K phosphorylates IRS1-4/*chico* triggering its internalization and subsequent proteasomal degradation. This feedback mechanism is reduced in pathological conditions, such as the Tuberous Sclerosis Complex syndrome (TSC), where cells carrying *tsc1* or *tsc2* mutations display an abnormal increase in size and exhibit constitutive phosphorylation of S6K (Saxton and Sabatini, 2017). As members of PI3Ks and TOR signaling are frequently activated in human tumors, they are attractive targets for cancer treatment.

#### *Myc* and Cell Competition

*MYC* is one of the most studied oncogenes, and its misexpression is associated with various tumor types including meningioma, Burkitt's lymphoma, medulloblastoma and hepatocellular carcinoma (Hsieh and Dang, 2016). *Drosophila* Myc is the sole fly member of the family of transcription factors that in mammals is composed of three genes (N-, L-, and *c-MYC*) (Gallant et al., 1996; Schreiber-Agus et al., 1997). Hypomorphic alleles of *myc* in flies are developmentally delayed and show a reduction in cell size resulting in smaller flies (hence the name of the mutant as *diminutive* = small) (Johnston et al., 1999), while null mutants die during larval stage (Pierce et al., 2004). Notably, ubiquitous expression of *myc* increases cell mass resulting in enrichment of genes encoding components of the nucleolus and of the ribosome; this evidence, concomitantly with Myc's ability to indirectly stimulate RNA pol I and III (Grewal et al., 2005; Hulf et al., 2005; Orian et al., 2005), contribute to revealing its role in the control of ribosomal biogenesis, thus mass and size. Myc activity is finely regulated, and while its expression is required at physiological levels during development, an excess of its activity triggers autonomous cell death and unbalanced growth (Grifoni and Bellosta, 2015). Therefore, Myc is strictly controlled both transcriptionally and post-translationally, where its protein stability is controlled by phosphorylation events downstream of RAS/ERK and GSK3β kinases with a signaling conserved in flies and mammals (Galletti et al., 2009; Parisi et al., 2011). Myc regulation of the cellular metabolic milieu is highly similar in *Drosophila* to the regulation found in tumor cells (DeBerardinis et al., 2008), indeed it was shown that in cells undergoing to a metabolic stress (starvation or competitive environment), expression of Myc switched their metabolism to increase glycolysis, glutaminolysis (Parisi et al., 2013; de la Cova et al., 2014; Hsieh et al., 2015), or lipid metabolism to favor survival by inducing autophagy (Parisi et al., 2013; Paiardi et al., 2017). Fascinatingly, these evolutionary functions of Myc to control mass and metabolism, resulted in the selective advantage of growth of epithelial cells described as cell competition and characterized in the monolayer epithelia composing *Drosophila's* imaginal discs (Johnston, 2014). Briefly, cells expressing Myc create a competitive environment and they grow at the expense of *wild-type* cells that are killed by non cell-autonomous apoptosis (de la Cova et al., 2004; Moreno and Basler, 2004). Myc cells thus behave as “winners” and they are able to repopulate the space of the dying “loser” cells that are killed by unidentified Myc-dependent mechanisms (Johnston, 2014). Myc-induced cell competition was also shown to be necessary in vertebrates to eliminate unfit cells (losers) during early embryogenesis (Claveria and Torres, 2016). More recently, evidence that sustains a central role for Myc-induced cell competition in the early steps of tumor formation have shown Myc present at high levels in cells surrounding the tumor near dying cells, potentially allowing the winner cells to expand and to eliminate the surrounding *wild-type* cells, thus establishing the first evidence of Myc involved in a tumor growth competitive environment (Johnston, 2014; Di Giacomo et al., 2017). Another form of cell competition is regulated by cell polarity genes *(lgl, scrib, dlg)* and by endocytic genes (such as *Rab5*). Cells mutant for these genes behave as losers and were eliminated by *wild-type* cells (Brumby and Richardson, 2003; Menendez et al., 2010); notably the expression of oncogenes in those loser clones provided them with super-competitive characteristics, i.e., *lgl* mutant cells over-expressing MYC send death signals to the adjacent *wild-type* proliferating cells (Froldi et al., 2010), suggesting the presence of another mechanism of cell competition driven by different growth forces working in combination with cell polarity genes and oncogenic signals.

## Organotypic *Drosophila* Cancer Models

### Gut Cancer

Similar to mammalian counterparts, the *Drosophila* adult gut is specialized in the digestion of food, the absorption of nutrients, and for controlling the defense response against infection (Tian et al., 2018). Based on these distinct functions, the *Drosophila* gut is composed of three parts: foregut, midgut, and hindgut. Among them, the midgut has a distinct architecture that resembles the digestive tract of vertebrates. The epithelium is a monolayer that is replenished by Intestinal Stem Cells (ISCs) that differentiate to either enteroblasts (EB) or pre-enteroendocrine cells (pre-EE), that then differentiate into absorptive enterocytes (EC) or secretory enteroendocrine cells (EE). Thanks to significant similarities in the physiology between the *Drosophila* gut and the intestine of vertebrates (Apidianakis and Rahme, 2011), *Drosophila* adult midgut epithelium has been used to study the contribution of signaling pathways (i.e., EGFR, Notch, Hedgehog, and Wg/Wnt) to Intestinal Stem Cells (ISCs) renewal (Jiang and Edgar, 2009; Biteau and Jasper, 2011; Jiang et al., 2011).

In vertebrates, the majority of sporadic cases of colorectal cancer and familial adenomatous polyposis (FAP) cancer syndrome are associated with activation of Wnt signaling (Bienz and Clevers, 2000). In humans, abnormal expression of Wnt in ISCs promotes adenoma formation, while deletions in mouse ISCs of the tumor suppressor *adenomatous polyposis coli* gene *APC* triggers the initial step of colon-adenoma formation (Barker et al., 2009), underlying the relevance of both mutations in this malignancy. In *Drosophila*, loss of the *Apc* gene, leads to the over proliferation of ISCs in the gut, resulting in loss of epithelial cell polarity, hyperplasia and epithelial overgrowth resembling that of intestinal adenomas induced by the loss of *APC* (Yu et al., 1999). Remarkably, the over-proliferation of the *Apc* ^−/−^ cells was rescued by *lof* mutation of *Ras* (Wang et al., 2013). On the contrary *Apc*^−/−^ cells expressing an active form of *Ras*^*v*12^ showed a malignant transformation including loss of cell polarity and invasive phenotype, highlighting the conserved functional cooperation between RAS and APC in controlling proper growth in the gut. In *Drosophila*, intestinal progenitors mutant for the *Apc* gene expand at the expense of the surrounding *wild-type* cells that die by apoptosis; because of this behavior these cells have been defined as “super-competitors” (Suijkerbuijk et al., 2016). *Apc* mutant cells exhibit higher Yki/YAP activity and increased JNK signaling, that was also detected at the border between *Apc*^−/−^ and *wild-type* cell; moreover, inhibition of apoptosis prevented *Apc* mutant cells from further expansion, suggesting that a competitive behavior in these cells is controlling *Apc* dependent tumor growth (Suijkerbuijk et al., 2016).

The JNK-Wg signaling is important to control the physiology and regeneration of intestinal cells, as ISCs damage leads to an overactivation of the JNK pathway and an increase in Wg ligand (Biteau et al., 2008; Cordero et al., 2012b). Wg activity in the enterocytes (ECs) indirectly drives the expansion of the ISCs by upregulating the JAK-STAT ligands Upd2 and Upd3, acting non-autonomously on ISCs proliferation (Tian et al., 2018). Moreover, activation of Wnt drives Myc upregulation in ISCs leading to non-autonomous upregulation of Upd3 in the ECs (Cordero et al., 2012a). Similarly, loss of *Apc1* in the midgut (ISCs) also results in JAK-STAT and EGFR pathway hyper-activation, and their removal suppresses the intestinal hyperplasia resulting from *Apc1* loss, revealing an underlying conserved signaling between flies and mammals that controls ISCs proliferation and gut homeostasis (Cordero et al., 2012a).

Another aggressive oncogene that is hyper-activated upon *Apc* loss, in mouse and human intestinal adenomas is the non-receptor tyrosine kinase *c-Src* (Yeatman, 2004). This proto-oncogene is amplified or activated in more than 20% of human tumors, and its activity has been demonstrated to play a central role in the formation of colorectal cancer (CRC). In mice, expression of *c-Src* increases in the proliferative progenitor cells of the “cripta” favoring hyperplastic adenoma formation (Cordero et al., 2014). In *Drosophila* the expression of *c-Src* orthologs (*Src42A* and *Src62B*) induces proliferation of the ISCs cells in *wild-type* animals, and reduction of their expression is sufficient to inhibit ISCs' hyper-proliferation of *Apc* mutant cells (Cordero et al., 2014). Notably, these results recapitulate an important part of the function of mammalian c-Src in the progenitor cells of the intestine during homeostasis and adenoma formation, suggesting a conserved role of this gene in flies in controlling proper ISCs proliferation.

Recently, *Drosophila* was also used to generate multigenic models of colon cancer using data from patients from The Cancer Genome Atlas. Interestingly, the outcomes of these models mimicked important properties of human cancers, and can be explored and used in chemical screens to find new combinations of cancer-relevant drugs (Bangi et al., 2016). Studies, using *Drosophila* models, to characterize intestinal human pathophysiology, revealed the high conservation between these species of the mechanisms underlaying colorectal tumorigenesis (Christofi and Apidianakis, 2013), and further revealed also the mechanisms that control the processes leading to bacterial-mediated inflammation (Lemaitre and Hoffmann, 2007).

### Brain Cancer

Meningioma are the most common intracranial tumors (Claus et al., 2005; Rogers et al., 2015) and frequently linked with mutations in the PI3K catalytic subunit p110α isoform encoded by the gene (*PI3KCA*), or in the *v-akt murine thymoma viral oncogene homolog 1* (*AKT1*) gene. Complex interactions were found between members of the PI3K/AKT/mTOR pathway and MAPK-, JAK/STAT, and Notch-1-mediated pathways that contribute to meningioma progression (El-Habr et al., 2014). Increased risk of meningiomas was associated also with neurofibromatosis type II syndrome, where mutations within the tumor suppressor gene *Suppressor of fused* (*SUFU*) was associated with hereditary meningiomas (Aavikko et al., 2012) and with medulloblastomas (Taylor et al., 2002). In *Drosophila* SUFU regulates Hedgehog (Hh) signaling (Ohlmeyer and Kalderon, 1998), with a similar function in humans, where loss of *SUFU* results in the aberrant activation of the Hedgehog (Hh) pathway (Aavikko et al., 2012).

Of all glioblastomas, the glioblastoma multiforme (GBM) is the most aggressive form of gliomas, accounting for approximately 50% of all glial tumors (Phillips et al., 2006). In GBM, Notch activity is associated with the control of Glioma Stem Cell (GSC), since its activity regulates asymmetric cell division and Notch unbalanced expression leads to uncontrolled growth and high malignancy (Mukherjee et al., 2016), Several studies demonstrate a role for Notch signal in controlling growth and stem cell maintenance of the brain also in flies (Song and Lu, 2011). Because of its conserved function, Notch pathway is now an important target for therapeutic intervention in brain cancer treatment (Yuan et al., 2015).

The current understanding of asymmetric cell division and its relation to tumorigenesis is largely derived from studies on *Drosophila* neuroblasts (NBs), where mutation of a single gene, *brain tumor* (*brat*), was shown to alter asymmetric stem cell division in larval development, and to generate massive neoplastic growth and enlarged adult brain formed entirely of neoplastic NBs (Caussinus and Gonzalez, 2005; Betschinger et al., 2006). Suppression of *brat* expression was used to establish a model of glioma stemness in *Drosophila*, where the upregulation of Notch, induced by reducing *brat*, was the critical node to maintain self-renewal and proper stemness (Mukherjee et al., 2016). This observation was also confirmed in glioblastomas where the human ortholog of *brat*, the tripartite motif-containing protein-3 (TRIM3), was shown to be necessary to suppress NOTCH1 signaling and to control stem cell activity during development to reduce tumor growth (Chen et al., 2014; Mukherjee et al., 2016). Glioma stem cells divide asymmetrically under the guidance of cell polarity complexes that control the proper apical and basolateral polarization and cell division, a process that was originally identified in *Drosophila* and later confirmed for the mechanism driving differentiation in human glia for members of the *Hugl-1/Llgl-1* complexes (Prehoda, 2009). We recently developed a neurogenic brain tumor model by impairing asymmetric cell division through the loss of function of *lethal giant larvae* (*lgl*) the *Drosophila* ortholog of *Hugl-1*, in the type II NBs of the central brain (Paglia et al., 2017). In our model, PI3K activation mimics PTEN loss of function and hampers Lgl localization at the apical membrane by aPKC cortical recruitment (Paglia et al., 2017). These data connect the function of *HUGL-1* in the maintenance of glioma stem cells with the loss of function of the tumor suppressor *PTEN* (Gont et al., 2013) and together with those in glioma (Read et al., 2009) show a conserved function for PI3K and EGFR overexpression in these tumors recapitulating many features of the neurogenic subtype of human glioblastoma. Inhibition of PI3K/Akt activity is currently used as a therapy in GBM (Zhao et al., 2017).

Other brain tumors such as oligodendrogliomas, that account for 10% of all cancers of the central nervous system, are characterized by mutations in the *capicua* (*cic*) gene (Bettegowda et al., 2011), a conserved transcriptional repressor that regulates MAPK effector genes downstream of receptor tyrosine kinase (RTK) (Simon-Carrasco et al., 2018). The development of correct animal model also for these tumors will be essential to develop specific treatments that can tackle these different brain tumors *in vivo*.

### The Paradigm for Angiogenesis

In the fruit fly, the circulatory system is open, the heart pumps the hemolymph into the body cavities and the exchange of gases takes place directly within the organs (Medioni et al., 2009). Moreover, *Drosophila* is equipped with a complex branched system of interconnected tubules that is responsible for the oxygen transport, the tracheal system, an organ that is comparable in structure and function to the circulatory system of mammals (Affolter et al., 2009). In *Drosophila*'s epithelia, the induction of clones bearing *lgl, Ras*^*V*12^ mutations identified how tumors are able to recruit vessels to oxygenate the growing mass (Grifoni et al., 2015; Calleja et al., 2016). These tumor cells showed ectopic expression of *Bnl* (*branchless*), the *Drosophila* homolog of Fibroblast Growth Factors (FGFs,), and suffered from oxygen shortage (hypoxia). In addition, it was observed a trans-differentiation of tumor cells into pseudo-tracheal cells with and the formation of new vessels, mimicking human FGF-mediated vascularization in cancer (Grifoni et al., 2015).

**Figure 3:**
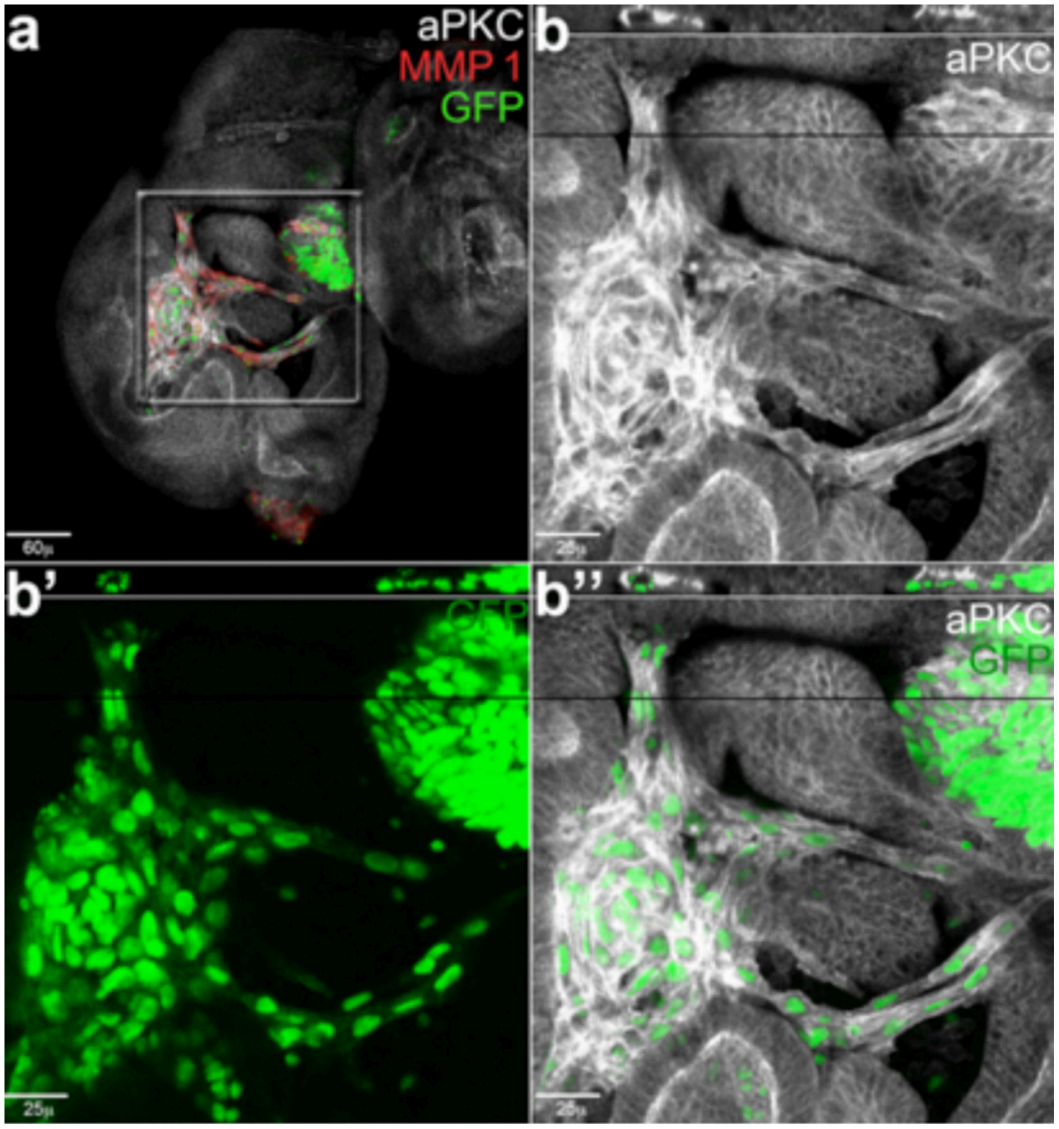
Cancer cells form branched and tubule-shaped structures (reproduced from Grifoni et al., 2015) with permission). **(a)** An imaginal wing disc bearing *lgl*^4^, Ras^V12^ clones induced in a wild-type background. **(b–b”)** Magnifications of the central region squared in **(a)**. Migrating tumor cells (GFP) are positive for the junctional marker aPKC (white) and secrete MMP1 (red). The reconstruction along the z-axis shown in the upper part of the magnified images reveals a tubule-shaped structure encircling a lumen, indicating these cells are forming tracheal-like structures.

Cell under hypoxia condition changes their cellular metabolism to favor growth, particularly in solid tumors (Pavlova and Thompson, 2016; Vander Heiden and DeBerardinis, 2017). Interesting studies in flies showed how reduction of the SCF (Skp/Cullin/F-box)-type ubiquitin ligase *Ago*, homolog of human *Fbw7*, increased tracheogenesis through up-regulation of the hypoxia-inducible transcription factor Sima/dHIF and of its target, the FGF ligand Bnl (Mortimer and Moberg, 2013). Fbw7 is known to inhibit tumor growth by targeting proteins to the proteasome pathways, and is mutated in a wide range of primary human cancers, this data suggests that its role as a tumor suppressor may be conserved also in the modulation of HIF-regulated angiogenesis in the tracheal system of the fly (Mortimer and Moberg, 2013). This process of neo-tracheogenesis is now considered a novel cancer hallmark in fly, which may help to explore the relation between angiogenesis and tumor growth in humans (Herranz et al., 2016; Figure 3).

### Lung Cancer

Lung cancer is a major cause of death in the world, and the standard therapeutic strategy used is chemotherapy because target therapies only decrease tumor growth and result in high toxicity. Recently, a new *Drosophila* lung cancer model was developed exploiting the tubular structure of the tracheal network (Levine and Cagan, 2016), and considered functionally and anatomically comparable to the vertebrate airways (Andrew and Ewald, 2010). Both in *Drosophila* and mammals, airways are formed by interconnected branches that depends on the secretion of Bnl/FGFs by the neighboring cells (Ghabrial et al., 2003; Grifoni et al., 2015). Using a binary system, *Ras*^*V*12^ was ectopically expressed specifically in the tracheal cells while downregulating *PTEN*, a negative regulator of the PI3K/AKT signaling (Hafen, 2004; Ortega-Molina and Serrano, 2013). As a result, the cells of the tracheal branches over-proliferated to form tumors that ultimately killed the animals (Levine and Cagan, 2016). This model was successfully used in a screen for chemical compounds approved by the Food and Drug Administration (FDA), which resulted in the identification of several compounds able to reduce cell over-proliferation and to improve tracheal physiological functions (Levine and Cagan, 2016), further highlighting the strong potential of the use of fruit fly models for cancer-related chemical screens.

### Prostate and Thyroid Cancer

The prostate is an exocrine gland of the male reproductive system responsible for the maturation and production of the seminal fluid, with its activity depending on androgens mostly produced by the testis. During organogenesis, the differentiation of the prostate's epithelium occurs along with that of stroma and depends on the complex coordination of many transcription factors and hormones that control the maturation of the quiescent organ (Toivanen and Shen, 2017). The adult prostate epithelium has a low turnover rate and its hyperplasia characterizes the majority of benign prostatic tumors. On the contrary, adenocarcinoma of the prostate is an aggressive tumor that rapidly progresses to a metastatic stage that can be partially blocked by androgen therapy (Shiao et al., 2016). Studies on flies' male accessory gland revealed many parallels with the physiology of human prostate epithelium (Wilson et al., 2017), i.e., a genetic screen using the *Drosophila* accessory gland identified genes that promote growth and migration of the secondary cells as homologs of genes expressed in human prostate cancer (Ito et al., 2014).

Like in human prostate, *Drosophila's* accessory gland presents a secondary layer of epithelial cells that continue to proliferate; this homology allowed the development of models that mimic tumors of endocrine origin, including human prostate and thyroid adenomas (Das and Cagan, 2013, 2018). For example, the multiple endocrine neoplasia type 2 (MEN2) syndrome, is characterized by different mutant-translocations involving the RET genes that result in multiple cancer phenotypes, including pheochromocytoma, parathyroid adenoma and the aggressive medullary thyroid carcinoma (MTC) (Das and Cagan, 2013). A recent study demonstrated that the papillary carcinoma of the thyroid (PTC), also caused by another genomic mutations of RET gene, can be profitably studied using the accessory gland of *Drosophila* to delineate and understand the mechanisms that characterize PTC in the context of the whole animal, including the relationship between tumor and normal cells in an environment that mimics tumor of endocrine origin in humans (Levinson and Cagan, 2016).

The prostate epithelium is characterized by the abundance of exosomes, microvesicles secreted from the endosomal multivesicular body (MVB) that fuse with sperm to modulate its activity and protect its homeostasis (Wilson et al., 2017). The exosomes are particularly relevant in cancer biology for their implication in tumor progression and survival, since they deliver survival factors, metabolites and miRNAs, that help creating a favorable microenvironment for cancer growth; in addition they also favor drug-resistance by activating mechanisms that favor the elimination of toxic chemicals such as chemotherapeutic products (Ruivo et al., 2017; Namee and O'Driscoll, 2018). Since the accessory gland has a similar structure as the prostate epithelium, characterized by the abundance of exosomes, it could be an optimal model to better study exosome biology in tumors of endocrine origins.

## Liquid Tumors

The signaling pathways regulating blood cell differentiation are conserved from *Drosophila* to humans (Lebestky et al., 2003; Jung et al., 2005). In addition, fly macrophages originate via self-renewal from progenitor cells localized in the lymph gland, a specialized hematopoietic organ that can be compared to the hematopoietic stem cell niche of the mammalian bone marrow (Krzemien et al., 2007; Mandal et al., 2007). These similarities with vertebrate hematopoiesis outline the utility of using fly models to elucidate the basic mechanisms of hematopoietic differentiation and homeostasis responsible for severe diseases, including leukemia. *Drosophila* has already been used to study Acute Myeloid Leukemia (AML), a widespread form of leukemia, and to identify the genes responsible for the disease. AML1 is a transcription factor, responsible for activating myeloid differentiation, which has a counterpart in the fly (Sinenko et al., 2010). In vertebrate tumors, the fusion of AML1 with the repressor ETO inhibits the differentiation of the multilineage progenitor cells, while their proliferation is activated, leading to AML1. Interestingly, AML1 fused with ETO in *Drosophila* also causes the inhibition of hematopoietic cell differentiation, confirming that the fly is a good genetic model to study the mechanisms that drive leukemia in humans (Osman et al., 2009; Sinenko et al., 2010). Myeloproliferative neoplasms (MPNs) have also been reproduced in the fly through gain-of-function mutations in the JAK pathway, finding a role for the downstream effector of the SWH pathway Yki in priming the expansion of *Drosophila* blood cells, which undergo malignant behavior following JAK activation (Anderson et al., 2017).

## Cancer and Immune System

Inflammation in tumor development acts as “tug and war” since it may promote survival of tumor cells by favoring angiogenesis, by reducing the natural immune responses and by altering responses to chemotherapeutic agents (Mantovani et al., 2008; Wu and Zhou, 2009). The inflammatory response of cancer cells has been attributed to a response of the immune system to eradicate the tumor, but it can also be seen as a way to provide growth and survival, as inflammation contributes to genomic instability by releasing cytokines and through production of reactive oxygen species (ROS) that may induce genetic and genomic alterations (Negrini et al., 2010). Normal cells detect and repair DNA damage, ensuring the maintenance of the correct number of chromosomes and tissue homeostasis, instead often cancer cells have increased mutation-rates leading to high chromosomal instability (CIN) that triggers aneuploidy and advances tumorigenesis (Negrini et al., 2010). Chromosomal instability is a process conserved also in *Drosophila*, and it was shown to contribute to the invasive behavior of epithelial cells, with a mechanism called “compensatory proliferation” activated to counteract CIN-induced cell death (Clemente-Ruiz et al., 2016; Benhra et al., 2018).

The mechanisms controlling cancer immune response are somehow conserved also in flies as studies in *Drosophila* have shown that infiltration of macrophages (called hemocytes) in cancer cells requires the activation of the JAK-STAT, JNK, TNF-α, and Toll/Imd/TLR signaling pathways (Bangi, 2013). Of particular interest is TNF-α that plays an important role in controlling apoptosis and the inflammation processes (Ham et al., 2016). TNF-α in tumors has distinct and overlapping functions to promote tumor growth and proliferation and to activate cell death, functions that are mainly mediated by the activation of TNFR1 that is ubiquitously expressed while TNFR2, mainly expressed in immune cells, is less well understood. Thus these opposite signaling pathways activated by TNF signals depend on the adaptor complexes recruited by the receptors and by the cellular context, and they may create a problem for the development of therapeutic strategies that target TNF signaling in tumors (Ham et al., 2016). In *Drosophila* the sole TNF-α, called Eiger (Egr), binds two receptors called Wengen (Kanda et al., 2002) and Grindelwald (Andersen et al., 2015), the latter shown necessary for the growth of *Ras*^*V*12^*/scribble*^−/−^ tumors (Andersen et al., 2015). An interesting mechanism links the possibility that ROS, induced by stress or local inflammation, triggers Egr expression in the hemocytes, to control JNK signaling, in a phenomenon called Apoptosis-Induced Proliferation (AIP), a sort of compensatory proliferative response of the epithelial cells that responds to cues from local “activated” hemocytes (Fogarty et al., 2016). Other studies highlighted the role of hemocytes in the interplay between inflammation and cancer, i.e., using a classic cancer model that recapitulates the hallmarks of epithelial cancer cells (*Ras*^*v*12^/*scribble*^−/−^), it was shown that cancer cells induce hemocyte's recruitment and proliferation *in vivo* by activating JNK signaling to cause the expression of JAK/STAT cytokines (Pastor-Pareja et al., 2008). Using a similar model it was shown that Egr expression was higher in the hemocytes derived from cancer animals, and that its activity was necessary to stimulate invasive migration of tumor cells (Cordero et al., 2010). On the contrary, Egr acts as a tumor suppressor to drive apoptosis in cancer cells upon activation of Toll/NF-κB signaling by the fat body (adipocytes) in response to the secretion of Egr by the circulating “activated” hemocytes (Parisi et al., 2014). Work using allograft transplantation experiments, identify also a function for the hemocytes in tumor initiation, that is independent on Eiger, but relays rather on the activation by external stimuli (i.e., CIN, abnormal growth) of JNK pathway and on the complex of non-autonomous and autonomous signals between tumor cells and those composing the tumor microenvironment; a similar mechanism has been proposed in vertebrates suggesting a conserved response for JNK signaling in fly to control initial tumor growth (Muzzopappa et al., 2017).

In summary, all these data suggest the existence of conserved mechanisms between the immune and tumor cells in flies that may recapitulate some of the most evolutionary conserved aspects described in cancer cells.

## Cancer and Lipid Metabolism, Obesity

In tumor biology, evidences highlight the relevance of lipid metabolism in influencing tumor growth (Katheder and Rusten, 2017; Weber et al., 2017). In this context, a recent role was identified for adipose triglyceride lipase (ATGL) whereby it hydrolyzes triacylglycerols into fatty acids (FAs) that may act as signaling molecules to induce growth both cell autonomously and in neighboring cells (Walther and Farese, 2012). The contribution of ATGL to cancer growth is controversial, indeed several studies showed that its depletion reduced proliferation in colorectal cancer cells and in non-small-cell lung carcinoma (Ou et al., 2014; Zagani et al., 2015), and in breast and pancreatic carcinoma its upregulation contributed to tumorigenesis (Grace et al., 2017; Wang et al., 2017). On the contrary, lack of *ATGL* favored pulmonary neoplasia in mice, and in few human tumors *ATGL* expression was found reduced highlighting the complex role of lipids in tumorigenesis (Al-Zoughbi et al., 2016). Cancer cells activate *de novo* lipogenesis by upregulation of key enzymes in lipid metabolism, some of which, such as AcetylCo-A Lyase (ACLY), AcetylCo-A Carboxylase (ACC) and Stearoyl-CoA desaturase-1 (SCD), are targets of pharmacological inhibitors to decrease cancer proliferation (Zaidi et al., 2012; Zu et al., 2013; Peck and Schulze, 2016; Stoiber et al., 2018). Recent work associated the mechanism of lypolysis with the induction of autophagy, a mechanism used by the cells to re-cycle part of their cytoplasm or cellular content to survive when nutrients are reduced (Dall'Armi et al., 2013). The relevance in cancer of the link between lipids and autophagy was shown when ATGL-mediated lipolysis in a peritumoral area, increased autophagy and tumor survival using a non-autonomous mechanisms (Martinez-Outschoorn et al., 2011; Gnerlich et al., 2013). Interestingly, we observed that Myc in *Drosophila* induced autophagy in the fat body and this was enough to enhance survival of the whole animals upon starvation (Parisi et al., 2013). We linked this effect with the ability of Myc to increase desat1, a Stearoyl-CoA desaturase-1 (SCD1) key enzyme in the synthesis of lipids, that we found co-expressed with Myc in human prostatic tumors (Paiardi et al., 2017).

Metabolic disorders and obesity are associated with cardiovascular disease and type II diabetes (T2D), however numerous cohort studies reported that overweight people are more likely to develop certain types of cancer including endometrial, breast, liver, and ovarian cancer (Cancer, 2012; Chen et al., 2012; Riboli, 2014; Wang and Xu, 2014; Dougan et al., 2015; Hirabayashi, 2016). Obese people have often increased levels of circulating hormones like insulin that has been associated to higher levels of IGF-1 in colon, kidney, prostate and endometrial cancer (Roberts et al., 2010; Gallagher and LeRoith, 2015). Another hormone, leptin, a cytokine produced by the adipocytes to control satiety in a signaling circuit of the brain, has also been found up-regulated in tissues from obese people, particularly in women post-menopause, and increased levels of leptin have been associated with higher incidence of breast and other tumors (Ray, 2018). The adipose tissue produces pro-inflammatory cytokines including IL-6, IL-8, IFNγ, and TNF-α among others (Scheller et al., 2011; Arango Duque and Descoteaux, 2014), and their over-production in fats from obese, activates the infiltration of macrophages into the adipose tissue inducing a low level of chronic inflammation or adipocyte tissue macrophage infiltration called ATM (Lafontan, 2014; Kuroda and Sakaue, 2017). This low level of inflammation increases the levels of ROS and induces DNA and protein damage that may increase the risk of cancer (Lafontan, 2014; Mraz and Haluzik, 2014). The role of the inflammatory response to combat infection and tissue injury, through the activation of the immune cells, is conserved also in *Drosophila's* circulating hemocytes (Lemaitre and Hoffmann, 2007), where most of the signals activated in the fat body results also in ROS production (Dionne, 2014; Vlisidou and Wood, 2015). Indeed, we showed, using a genetic model that harbors an inflammation state in the fat body of larvae that mimic ATM, that reduction of ROS, using exogenous anti-oxidants components like flavonoids and anthiocianins, decreased hemocyte's migration and JNK activation in the cells of fat body (Valenza et al., 2018), suggesting that the converging signaling between the fat body and hemocytes on lipid metabolism and ROS/cytokines in response to stress is conserved also in *Drosophila*.

## Cancer Stem Cells

Cancer stem cells (CSCs) have more features than tissue stem cells because they are able to initiate the tumor growth and fuel its maintenance and metastasis (Malanchi et al., 2011; Kreso and Dick, 2014). In addition, CSCs are highly resistant to conventional therapy, both radiation and chemotherapy, and they are responsible for the recurrence of disease (Mueller et al., 2009). Since the mechanisms underlying the ability of stem cells to support cancer progression are still unclear, *Drosophila* is convenient to use as it provides many tools for genetic and molecular investigations. Adult stem cells are required for tissue homeostasis and repair after injury and in adult flies, populations of stem cells are present in the posterior midgut, testis, and ovarian follicle rendering it again a good system to dissect these stem cell programs (Hou and Singh, 2017). *Drosophila* was used to better understand the functions of the centrosome and microtubule-organizing center (MTOC) in the division of stem cells (Tillery et al., 2018). *Drosophila* and mammalian stem cells are similar and they are regulated by homologous signals corroborating the use of the fly in this field of tumor biology. CSCs can arise from normal stem cells whose long lifespan favors the accumulation of genetic mutations responsible for the malignant phenotype. The progression from normal progenitors to stem-like cancer cells was first explored in leukemia, although nowadays we know that several solid tumors such as brain, breast, lung and colon cancer originate from cells with stem features (Krivtsov et al., 2006). Several *Drosophila* models of stem cell tumors are now available, and a drug screening was successfully carried out highlighting several compounds active on the signaling promoting cancer growth (Markstein et al., 2014).

## *Drosophila* Cancer Models for the Identification of Therapeutic Drugs

Therapeutic drug discovery requires chemical screening, a procedure allowing for the identification of potential new drugs. The spread of sequencing, automation, and miniaturization has made High Throughput Screening (HTS) the leading contributor to early-stage drug discovery. HTS consists of random screening of chemicals to find an affinity for a specific protein or biological activity characteristic of a disorder. Once identified *in vitro*, the compounds need to be validated *in vivo* to assess efficacy and toxicity during a long and expensive period of drug development. The high throughput assays depend on the existence of a specific target, assuming an in depth understanding of a disease that is not always available. Phenotype screening is an eligible option when the knowledge about the mechanisms underlying a disease process is not well defined. It is a process by which small molecules are screened for their effect on the phenotype in cells, tissue or whole animals, where a more physiological environment better describes the pharmacokinetics and toxicological effects of a drug. The great availability of genetic tools and the low cost of maintenance makes the fruit fly an ideal to model to study human diseases including cancer, in fact the fly has considerably contributed to understand tumor biology.

Chemical screens have been successfully performed in *Drosophila* for several disorders affecting the central nervous system, kidney and metabolism (Whitworth et al., 2006; Gasque et al., 2013; Hofherr et al., 2016), as well as for a type of thyroid cancer, the multiple endocrine neoplasia type 2A and 2B (MEN2) (Vidal et al., 2005). Regarding cancer, JAK- STAT, APC, Wnt, Notch and other signaling molecules, deeply characterized in *Drosophila* and shared with humans, are precious for cancer drug development. The availability of *Drosophila* models for multiple cancer types makes pharmacological screens possible against several drugs that aim to restrict proliferation and metastasis. The identification of anticancer compounds is possible using the adult fly, but also larvae, embryos and cells. The combined effect of anti-cancer drugs with radiation has been investigated in *Drosophila* larvae, producing similar findings to those observed in human cancer cells (Edwards et al., 2011). Moreover, *Drosophila* avatars, consisting of patient-specific tumors modeled in transgenic flies, are very promising for personalized medicine. *Drosophila* and other small model organisms are helpful to quickly analyze the mode of action of several active compounds *in vivo*, nevertheless mammalian models are indispensable in the successive phase of drug development to define important pharmacokinetic parameters such as absorption, distribution and metabolism.

## Discussion

The communication between tumor cells and their microenvironment is largely implicated in neoplastic growth, hence the substantial difficulty to recapitulate the features of malignant transformation in cellular systems. Cancer research needs *in vivo* investigations, and the use of model organisms contributes to answer this request. In this review we described most relevant approaches in *Drosophila*, used to explore cancer mechanisms and therapeutics that contribute to our understanding on tumor initiation and progression. In spite of some limitations, because of the anatomical differences between flies and humans, the use of *Drosophila*'s cancer models has been fundamental to understand some basic processes that regulate human cancers, such as the competitiveness of cancer stem cells (CSCs), the importance of tumor microenvironment, cancer cachexia, drug resistance and tumor-associated vasculogenesis, which was recently found to be functionally conserved in fly's cancer. Additional cancer hallmarks such as genomic instability, resistance to cell death, cell metabolism reprogramming, tumor-promoting inflammation and evasion from the immune system, have been studied and extensively characterized in *Drosophila*. Finally, although the evolutionary difference between *Drosophila* and humans certainly represents a restriction to the use of the fruit fly in drug discovery and development, phenotypic screenings have proven relevant to identify potential drugs that would elude the classic screens in the absence of targets. *Drosophila* is also offering a significant contribution to the investigation of organotypic cancers, since despite the evident differences at the macroscopic level, organ cells and functional units are usually well conserved at the biochemical and structural levels respectively. This conservation allowed to develop thyroid, lung, prostate, gut, brain and blood cancer models starting from the most characteristic genetic lesions found in the same human cancers. These models, as described in the review, are greatly helping in dissecting the contribution of specific molecular pathways to the final cancer phenotype. Given the heterogeneous nature of mammalian solid cancers, new strategies are being developed to decipher cancers at single-cell resolution. The international *Drosophila* community has always been engaged in the development of novel, sophisticated genetic tools, which allowed in the last 30 years to revolutionate functional gene analysis. For this reason, we anticipate that the use of the fruit fly will move fast into the field of precision medicine, contributing to seminal findings in this new era of cancer research.

## Author Contributions

All authors listed have made a substantial, direct and intellectual contribution to the work, and approved it for publication.

### Conflict of Interest Statement

The authors declare that the research was conducted in the absence of any commercial or financial relationships that could be construed as a potential conflict of interest.
